# Myelin 2′,3′-Cyclic Nucleotide 3′-Phosphodiesterase: Active-Site Ligand Binding and Molecular Conformation

**DOI:** 10.1371/journal.pone.0032336

**Published:** 2012-02-29

**Authors:** Matti Myllykoski, Arne Raasakka, Huijong Han, Petri Kursula

**Affiliations:** 1 Department of Biochemistry and Biocenter Oulu, University of Oulu, Oulu, Finland; 2 Centre for Structural Systems Biology, Helmholtz Centre for Infection Research (CSSB-HZI), German Electron Synchrotron (DESY), Hamburg, Germany; 3 Department of Chemistry, University of Hamburg, Hamburg, Germany; Semmelweis University, Hungary

## Abstract

The 2′,3′-cyclic nucleotide 3′-phosphodiesterase (CNPase) is a highly abundant membrane-associated enzyme in the myelin sheath of the vertebrate nervous system. CNPase is a member of the 2H phosphoesterase family and catalyzes the formation of 2′-nucleotide products from 2′,3′-cyclic substrates; however, its physiological substrate and function remain unknown. It is likely that CNPase participates in RNA metabolism in the myelinating cell. We solved crystal structures of the phosphodiesterase domain of mouse CNPase, showing the binding mode of nucleotide ligands in the active site. The binding mode of the product 2′-AMP provides a detailed view of the reaction mechanism. Comparisons of CNPase crystal structures highlight flexible loops, which could play roles in substrate recognition; large differences in the active-site vicinity are observed when comparing more distant members of the 2H family. We also studied the full-length CNPase, showing its N-terminal domain is involved in RNA binding and dimerization. Our results provide a detailed picture of the CNPase active site during its catalytic cycle, and suggest a specific function for the previously uncharacterized N-terminal domain.

## Introduction

The myelin sheath is a crucial component of the vertebrate nervous system, speeding up nerve impulses and supporting axonal integrity. The multilayered myelin membrane is rich in lipids and low in water, with a number of myelin-specific proteins. 2′,3′-cyclic nucleotide 3′-phosphodiesterase (CNPase), one of the proteins predominantly expressed in the myelin sheath, represents 4% of total protein in central nervous system myelin [1]. It is localized in the non-compacted regions of myelin, where it is a quantitatively major protein. CNPase has been implicated as an autoantigen in multiple sclerosis [2], [3], and mice deficient in CNPase suffer progressive axonal degeneration leading to premature death [4].

CNPase belongs to the 2H phosphoesterase superfamily [5], [6], which is characterized by the presence of two conserved HxT/Sx motifs (x denoting a hydrophobic residue) in the active site. The 2H family members exhibit a 3′-phosphodiesterase activity towards 2′,3′-cyclic nucleotides, oligonucleotides, or RNA. The 2H phosphoesterases have large sequence variations, but the core fold and the active site residues are conserved [6].

CNPase contains an N-terminal domain very distantly related to P-loop containing nucleoside triphosphate hydrolases [7], [8] and a C-terminal phosphodiesterase domain [9]. The N-terminal domain is poorly characterized, and its function is unknown to date. Structural data exist for the phosphodiesterase domain from different species [5], [10], [11], but no structures have been solved for 2H phosphoesterases complexed with substrates or products, or for full-length CNPase. At its very C-terminus, CNPase has an isoprenylation site, linking the protein to the membrane [12]. Thus, the active site of CNPase lies very close to the membrane surface.

Even 50 years after its initial characterization [13], CNPase is enigmatic with regard to its physiological function. During recent years, possible functions have, little by little, been elucidated. The properties of CNPase, including membrane attachment [14], interactions with cytoskeletal proteins [15]–[17], and binding to RNA [18], have implied that CNPase could be involved in RNA trafficking, splicing, or metabolism in the myelinating glial cell. CNPase has also been suggested to regulate the maturation of oligodendrocytes and the expression of myelin genes [19]. For example, mRNA for the major myelin protein MBP is transported to oligodendrocyte processes [20], and CNPase could play a role in its processing or degradation.

Based on its activity and homology to other enzymes, CNPase might function in RNA splicing. When an intron is spliced from immature tRNA, a 2′,3′-cyclic phosphate group is formed [21]. In yeast, the subsequent ligation is performed by a single multifunctional tRNA ligase, Trl1. Trl1 contains CNPase activity to heal the cyclic nucleotide, polynucleotide kinase activity to phosphorylate the 5′-OH group of the 3′-exon with phosphate from GTP, and ATP-dependent ligase activity to seal the exons [22], [23]. In yeast, Trl1 is necessary for complete tRNA splicing and, thus, essential for the cells [24]. Rat CNPase is able to complement the phosphodiesterase, but not kinase, activity of yeast Trl1 [25]. Thus, CNPase is able to act as a functional entity in the yeast tRNA splicing mechanim, but its possible role in the corresponding mechanism in vertebrates in unclear.

In this study, crystal and solution structures of the CNPase catalytic domain were solved in the presence of active site ligands. The structural data can be used to elucidate the fine details of the enzymatic phosphodiesterase activity in CNPase. We also provide data on the possible functions of the CNPase N-terminal domain.

## Results and Discussion

### Overall structure of mouse CNPase catalytic domain

We solved the crystal structure of the C-terminal catalytic phosphodiesterase domain of mouse CNPase in the presence of active-site ligands, including reaction products (Table 1,2). Remarkably, no crystal structures of 2H phosphoesterases with bound substrates or products have been reported earlier.

**Table 1:** Crystallization conditions and treatments.

Crystal form	Well solution	Additives	Soaking details	Cryoprotection
Orthorhombic citrate complex	0.1 M sodium citrate pH 3.9, 26% PEG 3350	10 mM 2′,3′-cAMP	-	0.1 M sodium citrate pH 3.5, 25% PEG 3350, 20% glycerol, 10 mM 2′,3′-cAMP
Monoclinic citrate complex	0.1 M sodium citrate pH 3.3, 26% PEG 3350	10 mM 2′,3′-cNADP	-	0.1 M sodium citrate pH 3.5, 25% PEG 3350, 20% glycerol, 10 mM 2′,3′-cNADP^+^
Sulphate complex	50 mM sodium citrate pH 3.5, 2.9 M (NH_4_)_2_SO_4_, 10% PEG 550 MME	-	-	-
2′-AMP complex	50 mM sodium citrate pH 3.5, 30% PEG 3000	-	50 mM sodium citrate pH 3.5, 35% PEG 1500, 100 mM 2′,3′-cAMP (4 h)	-
NADP^+^ complex	50 mM sodium citrate pH 3.5, 30% PEG 3000	-	50 mM sodium citrate pH 3.5, 35% PEG 1500, 100 mM 2′,3′- cNADP^+^ (25 min)	-

**Table 2:** Data processing and structure refinement.

	Dataset				
Active site ligand	Citrate	Citrate	Sulphate	NADP^+^	2′-AMP
PDB entry	2xmi	2y1p	2y3x	2ydb	2ydd
Data collection and processing:					
Beamline	BESSYBL 14.1	BESSYBL 14.1	BESSYBL 14.1	MAX-LAB I911-2	MAX-LAB I911-2
Wavelength (Å)	0.91841	0.91841	0.91841	1.03796	1.03796
Unit-cell parameters (Å)	a = 41.6 b = 47.1 c = 107.1	a = 42.0 b = 46.9 c = 54.9	a = b = 82.4 c = 86.3	a = 41.7 b = 46.8 c = 106.9	a = 41.4 b = 46.8 c = 106.6
(°)	α = β = γ = 90	α = γ = 90 β = 95.5	α = β = 90 γ = 120	α = β = γ = 90	α = β = γ = 90
Space group	P2_1_2_1_2_1_	P2_1_	P3_1_	P2_1_2_1_2_1_	P2_1_2_1_2_1_
Resolution range (Å)*	35–1.74 (1.79–1.74)	26–1.82 (1.87–1.82)	35–2.1 (2.15–2.10)	25–2.15 (2.21–2.15)	20–2.4 (2.46–2.40)
Observed/unique reflections	154941/22280	71653/19148	147089/38236	32782/11501	39972/8420
R_sym_ (%)	6.6 (94.0)	7.1 (67.5)	4.1 (55.7)	5.0 (41.6)	9.9 (93.9)
<I/σI>	17.7 (2.1)	14.2 (2.0)	20.0 (2.5)	13.2 (2.5)	13.0 (1.8)
Completeness (%)	99.9 (99.7)	99.7 (99.7)	99.8 (99.8)	96.2 (90.2)	98.2 (99.5)
Redundancy	7.0 (6.1)	3.7 (3.7)	3.9 (3.8)	2.9 (2.4)	4.7 (4.8)
Refinement:					
R_cryst_ (%)	17.8	15.8	19.0	19.7	20.7
R_free_ (%)	20.9	19.9	22.8	25.6	26.9
Wilson *B* factor (Å^2^)	31.4	27.7	49.3	43.5	45.3
Average *B* factor (Å^2^)	36.5	39.0	72.0	50.0	68.5
R.m.s.d. bond length (Å)	0.006	0.009	0.015	0.006	0.010
R.m.s.d. bond angle (°)	0.9	1.1	1.6	1.0	1.3
Missing residues	158–159, 210–212	158–160	A: 158–163, B: 158–159, 209–212, C: 158–163. 208–212	158–162, 209–211	158–162, 207–212
Molprobity analysis:					
Clashscore (percentile)	7.3 (85th)	12.5 (61st)	9.3 (88th)	11.9 (79th)	3.6 (99th)
Ramachandran plot					
Favoured (%)	99.5	96.8	98.3	97.6	97.6
Disallowed (%)	0.0	0.0	0.0	0.0	0.0
Molprobity score (percentile)	1.40 (95th)	1.81 (78th)	1.84 (89th)	2.02 (82nd)	1.78 (97th)

* numbers in parentheses correspond to the highest-resolution shell.

**Figure 1:**
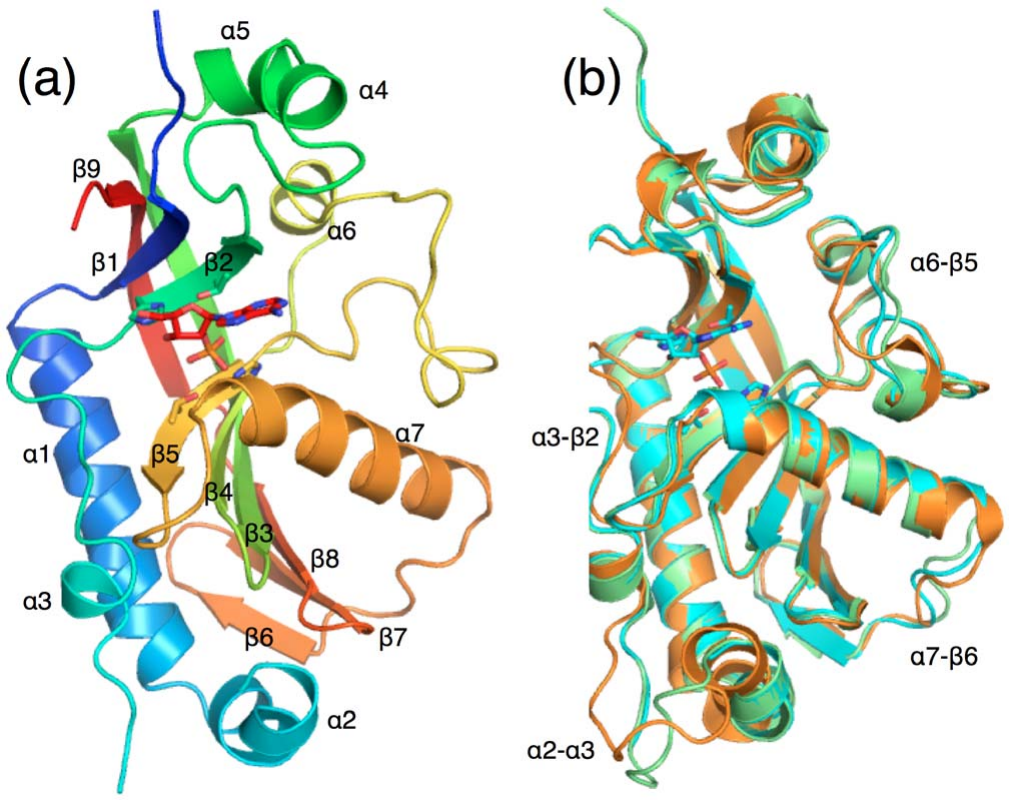
The overall structure. (a) Crystal structure of the mouse CNPase phosphodiesterase domain. The side chains of the conserved HxTx motifs and the bound 2′-AMP in the active site are shown as sticks. The protein is colored from blue (N-terminus) to red (C-terminus), and secondary structure elements are labelled. (b) Superposition of the three different crystal forms of mouse CNPase determined in this study, indicating flexibility mainly in 4 loops.

The overall structure can be divided into two lobes, between which the active-site cleft is located (Figure 1A). The small lobe contains both the N- and C-termini in close proximity, and is comprised of four β strands, three short helices, and a flexible loop between residues 290 and 308 (α6-β5 loop). The large lobe contains six antiparallel strands, two long and two short helices, and three long loops (α2-α3, α3-β2, α7-β6). Differences between the crystal structures of murine CNPase obtained in this work are found in these flexible loops (Figure 1B).

### The CNPase active site

**Figure 2:**
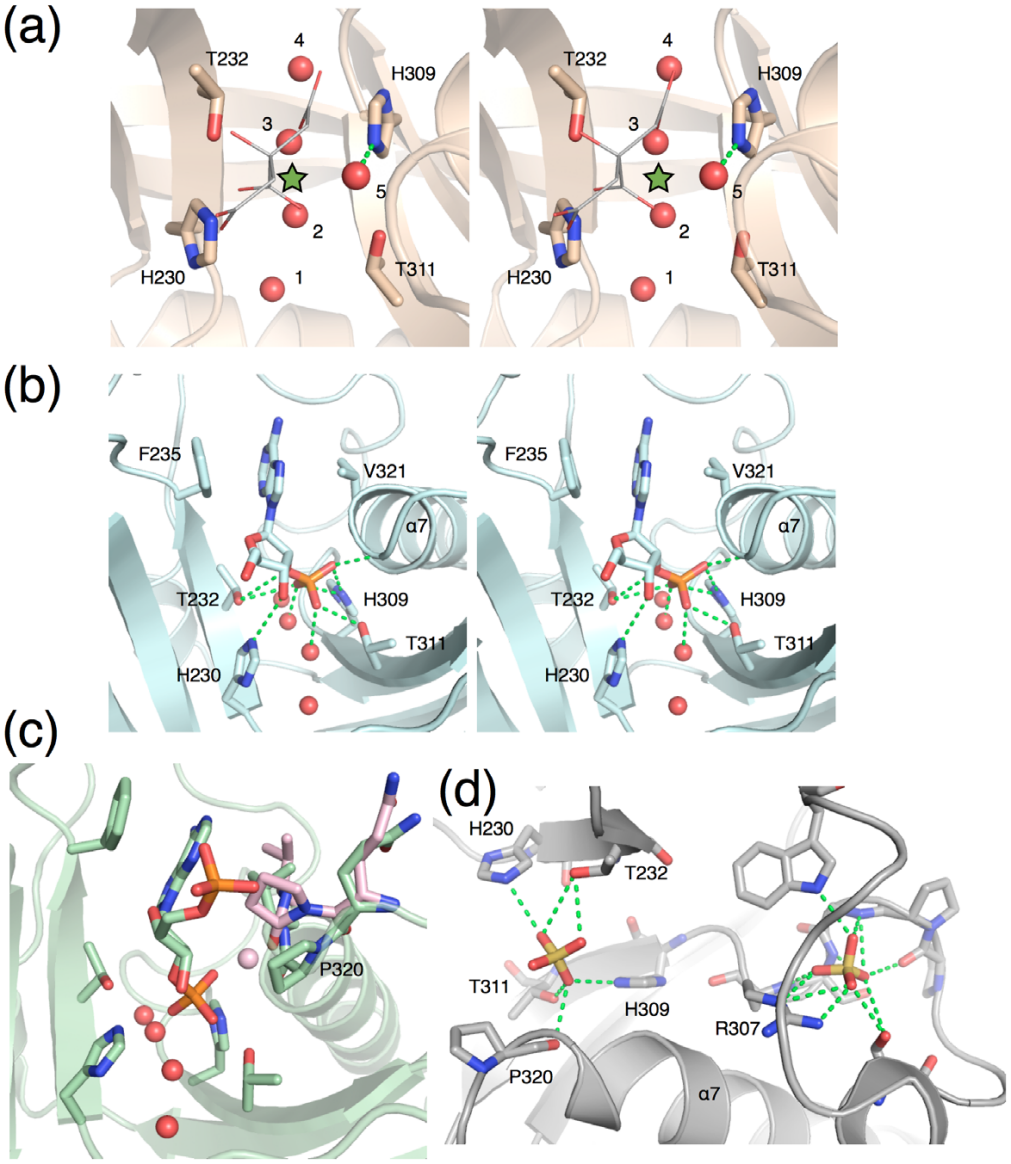
Details of the active site. (a) An overall view of the CNPase active site. The apparent 2-fold symmetry axis of the active site is indicated by the green asterisk. The catalytic residues and 5 ordered water molecules are labelled. In this structure, the active site is occupied by a citrate molecule. (b) Stereo view of the binding mode of 2′-AMP to mouse CNPase. (c) Complex with NADP^+^. The double conformation of the region around Pro320 is also shown; the conformation in green corresponds to the one with the ligand, and the pink one to the conformation without bound ligand. (d) CNPase complex with two sulphate molecules. One sulphate resides in the active site (left) and the other binds to the α6-β5 loop (right).

The active site of CNPase contains two HxTx motifs at residues 230–233 and 309–312, at the bottom of a narrow cleft between the two lobes (Figure 2). This cleft is enveloped from one side by Pro320, Val321, and Arg307 and from the other side by Phe235, Leu167, and Tyr168. Conserved ordered water molecules are present in the active site (Figure 2A), which we number as waters 1–5 for clarity and consistency. Waters 1–4 line the bottom of the active site cleft, while water 5 was suggested to be a nucleophilic water in the reaction mechanism [11]. Interestingly, the strands β2 and β5, on which the HxTx motifs lie, do not interact through direct backbone hydrogen bonds; instead, the conserved water molecules 1–4 mediate hydrogen bonds between the two adjacent strands. This arrangement may bring additional flexibility to the core of the active site.

The core catalytic site, with the two HxTx motifs and 4 water molecules, has a pseudo-twofold symmetry (Figure 2A). However, the symmetry does not extend beyond this region, and substrate recognition is asymmetric. This is important for CNPase specificity, selectively cleaving the 3′-phosphodiester bond from 2′,3′-cyclic substrates.

No structural information has been available for nucleotide complexes of CNPase or its closest homologues. NMR studies on RICH [10] and CNPase [5] have indicated roles for Phe235 and Val321 – and its nearby residues at the N-terminus of helix α7 - in recognition of the nucleotide, while the phosphate complex [11] indicated the position of catalysis. The detailed binding modes of nucleotide ligands have not been elucidated. Here, we determined crystal structures of CNPase with 4 different active-site ligands (Figure 2), including 2′-AMP, NADP^+^, sulphate, and citrate; 2′-AMP and NADP^+^ are reaction products formed during the experiment from the respective 2′,3′-cyclic substrates. With the combination of these structural data, we can now get a much deeper insight into catalysis by CNPase than previously possible.

### Binding mode of reaction products

Two structures were solved in the presence of a reaction product in the active site; they resulted from soaking the crystals in excess of the *in vitro* substrates 2′,3′-cAMP and 2′,3′-cNADP^+^. In both structures, the cyclic bond has been cleaved, and a 2′-product is located in the active site (Figure 2B,C, Figure S1); the nucleotide occupies a similar position in both structures. The treatment with 2′,3′-cAMP resulted in a well-defined molecule of 2′-AMP in the active site. The crystal soaked in 2′,3′-cNADP^+^ indicated a lower occupancy of the reaction product NADP^+^ in the active site; the 2′-AMP moiety and the 5′-phosphate were, however, visible in the electron density. The 2′-AMP complex will be mainly discussed here, due to its high occupancy and strong electron density.

The 2′-AMP is tightly bound in the narrow active-site cavity. The adenine base makes stacked π-π interactions with the side chain of Phe235, while both of its rings are involved in CH-π hydrogen bonds with Val321 (Figure 2B). The ribose moiety interacts with CNPase mainly through van der Waals interactions to Pro320, Val321, and Tyr168. The 3′-OH group (the leaving group in the phosphodiesterase reaction) is H-bonded to the Nε2 atom of His230. A H-bond also exists between the side chain hydroxyl of Thr232 and the phosphoester oxygen.

Most polar interactions between 2′-AMP and the active site are mediated by the phosphate group (Figure 2B). Two of its oxygen atoms are coordinated by water molecules 2 and 3 at the center of the active site; one of these oxygens is also H-bonded to Thr311 and the other one to His309. The third free oxygen of the phosphate is in van der Waals contact with the backbone carbonyl group of Pro320 – the geometry is suboptimal for hydrogen bonding. It is also H-bonded to the Nε2 atom of His309. This is most likely the position of the nucleophilic water prior to the reaction, and corresponds to the location of a water molecule (water 5) in other structures. The 2′-nucleotide being a mimic of the end of a processed RNA molecule, the possible direction of entrance by the RNA molecule can be inferred from the binding mode of the nucleotide (see below).

In the NADP^+^ complex, residues 319–321 of CNPase are in a double conformation related to ligand binding (Figure 2C). These residues are at the N-terminus of the α7 helix, and this long helix provides a positive electrostatic environment in the vicinity of the active site through its helix dipole moment. A role for the flexibility of the N-terminus of helix α7 has also been indicated by the fact that a G324A mutation in rat CNPase affects both substrate binding and catalytic efficiency [5]. The first conformation is present in all of the structures. We believe this conformation to be linked to ligand binding. In the second conformation, Pro320 is moved away, and the nucleophilic water is present. Residue 320 is not conserved between species, but its carbonyl group, interacting with the nucleophilic water and the product phosphate, is apparently important. Val321 and Phe235 sandwich the nucleotide base, and Pro320 interacts with the active site ligands and the nucleophilic water. Kozlov *et al*. reported chemical shift perturbations in residues Val321-Gly324 upon ligand binding [5]. It is likely that this conformational flexibility of the N-terminus of helix α7 is related to the reaction cycle of the enzyme.

### Complexes with sulphate and citrate

In CNPase crystallized in concentrated ammonium sulphate, two sulphate molecules are located in the vicinity of the catalytic site. The active-site sulphate mimics the substrate phosphate group and interacts with all 4 conserved residues of the two HxTx motifs and the two central water molecules in the catalytic site through H-bonds (Figure 2D), being also in contact with the backbone carbonyl of Pro320 and bulk solvent. The second sulphate stabilizes the long α6-β5 loop. Arg307, between the two sulphate binding sites, adopts a different conformation in the sulphate complex, turning away from the active site. The significance of the α6-β5 loop is not known, but the detection of a sulphate-binding site and the different conformations seen for the loop suggest it could be relevant in CNPase function.

The structures with citrate in the active site were determined at the highest resolutions. The primary interaction of the citrate with the active site comes from the central carboxyl group, bound to His230 and Thr232 (Figure 2A). The nucleophilic water 5 is bound to the central and terminal carboxyl groups of citrate and to His309 and the carbonyl oxygen of Pro320.

### Comparison to homologous structures

The structurally characterized members of the 2H phosphoesterase family can be divided into two groups (Table 3, Figure S2). The first group contains mammalian CNPases and goldfish RICH. The more distant group contains bacterial and archaeal 2′-5′ RNA ligases [26]–[28], a plant cyclic nucleotide phosphodiesterase (CPDase) [29], and a human protein kinase A anchoring protein (AKAP18) [30].

**Table 3:** CNPase structural homologs, for which experimental structures are available.

Protein	Organism	PDB id (reference)	Active-site ligand	Rmsd (# residues)	Sequence identity (%)
CNPase	*Homo sapiens*	1woj [11]	Phosphate	0.8 (200)	81
CNPase*	*Rattus norvegicus*	2ilx [5]	-	2.2 (194)	85
RICH*	*Carassius auratus*	2i3e [10]	-	2.5 (187)	43
2′-5′ RNA ligase	*Thermos thermophilus* HB8	1iuh [28]	-	2.9 (106)	25
2′-5′ RNA ligase	*Pyrococcus horikoshii*	1vgj [26]	-	2.8 (106)	14
CPDase	*Arabidopsis thaliana*	1fsi [29]	Sulphate	3.2 (107)	14
2′-5′ RNA ligase*	*Pyrococcus furiosus*	2fyh [27]	-	3.0 (104)	14
Putative 2′-5′ RNA ligase	*Bacillus subtilis*	2d4g (-)	-	3.5 (104)	13
AKAP18 central domain	*Homo sapiens*	2vfk,2vfl,2vfy [30]	5′-AMP,-,5′-CMP	3.2 (106) (2vfk)	10
ATU0111 (unknown function)	*Agrobacterium tumefaciens str. C58*	2fsq (-)	Acetate	3.1 (109)	16

* NMR.

**Figure 3:**
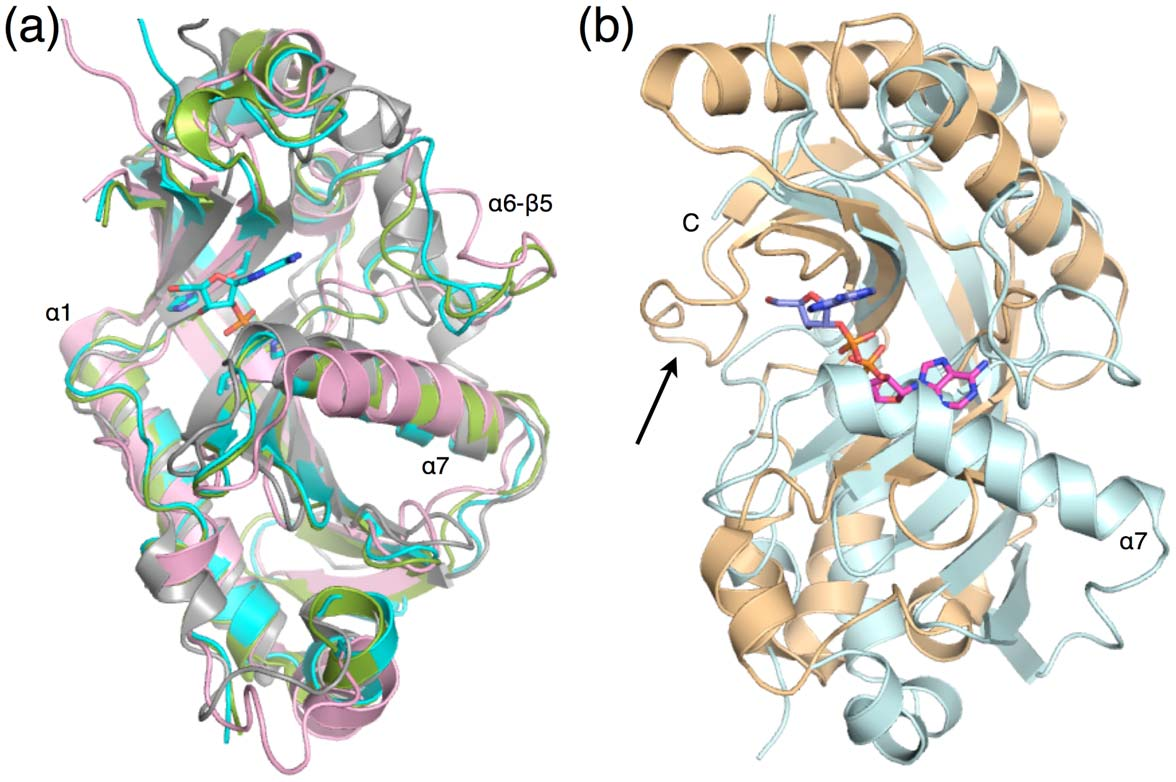
Comparisons to structures of 2H family members. (a) Superposition of the CNPase/RICH structures. Colouring: mouse, light blue; human, green; rat, pink; goldfish RICH, gray. Note the conservation of the α7 helix in all structures; the 2′-AMP from the mouse CNPase active site is also shown. (b) Superposition of mouse CNPase (light blue) and human AKAP18 (orange), the two nucleotide-bound complexes of 2H family members. The C-terminus and the α7 helix of CNPase are labelled, and the covering C-terminal loop of AKAP18, present in non-CNPase 2H family members, is indicated by an arrow. The 2′-AMP in CNPase is in blue and the 5′-AMP in AKAP18 in magenta. Note that only the phosphate groups of the nucleotide ligands overlap.

The core structure of the mouse CNPase catalytic domain is very similar to the human enzyme and RICH [10], [11]. The largest difference between the mouse and human CNPase is in the α6-β5 loop; this is also a region of least sequence conservation between the two species. The rat CNPase NMR solution structure also shows most differences in the loops; in addition, helix α7 is in a slightly different orientation compared to the crystal structures, opening up the active site (Figure 3A).

Also in more distant family members, the essential active site residues are similarly located. The catalytic cleft, symmetrically formed on top of antiparallel strands, is structured in a similar fashion. The long helix α1 is similar in all compared structures. There are, however, interesting differences - possibly affecting substrate binding - between CNPases/RICH and the other 2H phosphoesterases.

A major difference is that the long helix α7 is not conserved in non-CNPase structures (Figure 3B, Figure S2). The N-terminus of the α7 helix borders the CNPase active site, interacts closely with the substrate and the nucleophilic water, and effectively blocks the entrance of large molecules from this direction. The absence of α7 may create a binding pocket for large substrates in some of the other RNA-processing enzymes within the family, and it is possible that these enzymes also have differences in their respective catalytic mechanisms.

Another major difference is the presence of a covering region on the opposite side of the active site in non-CNPase structures (Figure 3B). This cover is formed by a β-hairpin segment, which in sequence alignments, is located after the C-terminal end of the CNPase folded domain (Figure S2).

Taken together, the CNPase active site cleft is more open towards the His230 side of the cleft, as opposed to the 2′-5′ RNA ligase, CPDase, and AKAP18, which are more open towards the His309 side. The crystal structure of the 2H family domain of AKAP18 has been solved with bound 5′-AMP and 5′-CMP, although no enzymatic activity has been observed [30]. Correlating with the differences outlined above, the active-site nucleotide binding modes in AKAP18 and CNPase are distinct, with the phosphate group being bound to the HxTx motifs in both proteins (Figure 3B). The results further indicate that in 2H family members, the ligand binding mode is conserved only for the reactive phosphate moiety, and arrangements around the active site in the protein, concerning both secondary structure elements and flexible loops, significantly affect the actual binding mode of the full substrate, further having effects on reaction specficity.

### Solution studies identify open and closed conformations

**Figure 4:**
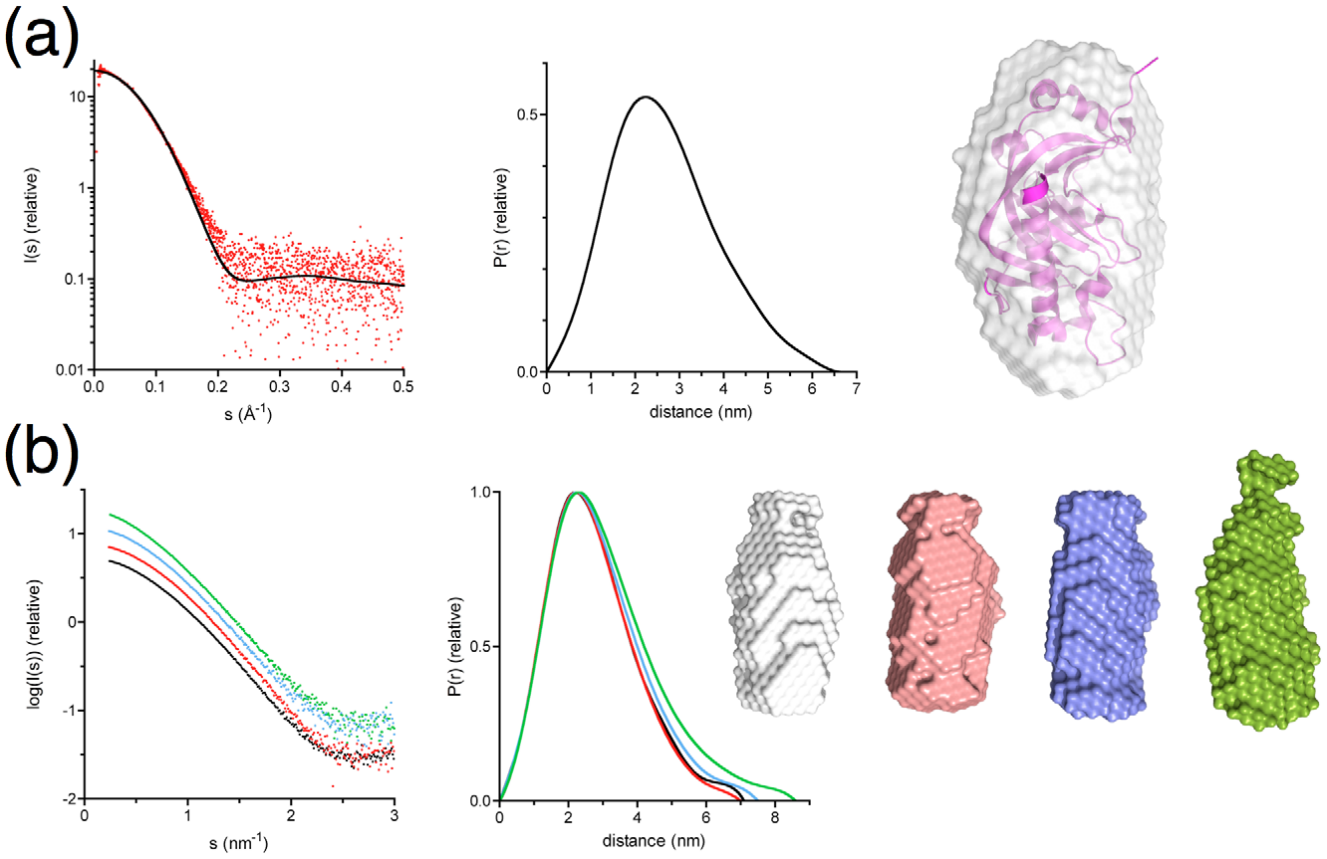
Conformation of the CNPase catalytic domain as detected by SAXS. (a) Left: Experimental scattering curve for the CNPase catalytic domain (red dots) overlaid with the theoretical scattering curve computed using the crystal structure (black line). The Guinier plot corresponding to this scattering curve is shown in Figure S4. Middle: Distance distribution function. Right: Averaged *ab initio* model of the phosphodiesterase domain (white surface) superimposed with the crystal structure (magenta). (b) SAXS analysis of extended CNPase catalytic domain constructs – left: scattering curves, middle: distance distribution functions. Black: the crystallized catalytic domain; red: construct cat-C; blue: construct N-cat; green: construct N-cat-C. The scattering curves are slightly displaced in the y direction for easier viewing: the corresponding Guinier plots are shown in Figure S4. Right: *Ab initio* models of the catalytic domain (gray), cat-C (pink), N-cat (blue), and N-cat-C (green). The results indicate that the N- and C-terminal extentions alone affect the structure little, while an extended tail is formed when both of them are present.

Normal mode analysis (Figure S3) of the crystal structures always indicated a very strong collective mode related to the opening of the active-site cavity between the two lobes. The structure of the CNPase catalytic domain in solution was studied by small-angle X-ray scattering (SAXS), in order to observe possible conformational changes. The catalytic domain was monomeric under all employed conditions, with a very similar overall shape to that in the crystal structure (Table 4, Figure 4). Crystallization was only successful with sulphate or citrate present. SAXS analysis in the presence of citrate also shows a compact structure, very similar to the crystal structure; analysis of the concentration dependency indicated that citrate reduced the attractive forces between protein molecules (Figure S4). The result explains the crystallization behaviour of CNPase. The fully active overall conformation of CNPase in solution may, however, be more open than that seen in crystal structures, as all the crystals only grew in the presence of citrate or sulphate.

**Table 4:** Active-site ligand-dependent solution properties of the CNPase catalytic domain, as determined by SAXS.

Sample	D_max_ (nm)	R_g_ (nm)	Volume (nm^3^)	I(0) (relative)
CNPase	6.8	2.06	48.2	19.7
Crystal structure envelope*	6.8	1.84	30.8	-
Catalytic#	7.1	2.13	44.4	
cat-C	6.8	2.09	46.0	
N-cat	7.0	2.20	48.3	
N-cat-C	8.6	2.42	52.2	

* as calculated using CRYSOL [60].

# samples starting from this line were measured at a different beamline.

In full-length CNPase, the catalytic domain is followed by 22 C-terminal residues that contain the membrane anchoring site. A structure of CNPase with the very C-terminus is not available, but it is unlikely that this tail is folded [12], [31]. Our SRCD studies indicated that a palmitoylated peptide comprising the C-terminus is unfolded in membrane-mimicking conditions [31]. We further studied the solution conformations of extended versions of the catalytic domain by synchrotron SAXS (Table 4, Figure 4C,D, Figure S4). The presence of the C-terminal tail (construct cat-C) did not significantly affect the conformation of the catalytic domain in solution. For the N-terminally extended catalytic domain (N-cat), overall dimensions remained similar, with an increased radius of gyration. Only when both extensions were included (N-cat-C), did the molecule become significantly more elongated. The result highlights the short distance that must remain between the active site and the membrane surface, to which the C-terminus is anchored *in vivo*.

### The N-terminal domain interacts with RNA and mediates dimerization

**Figure 5:**
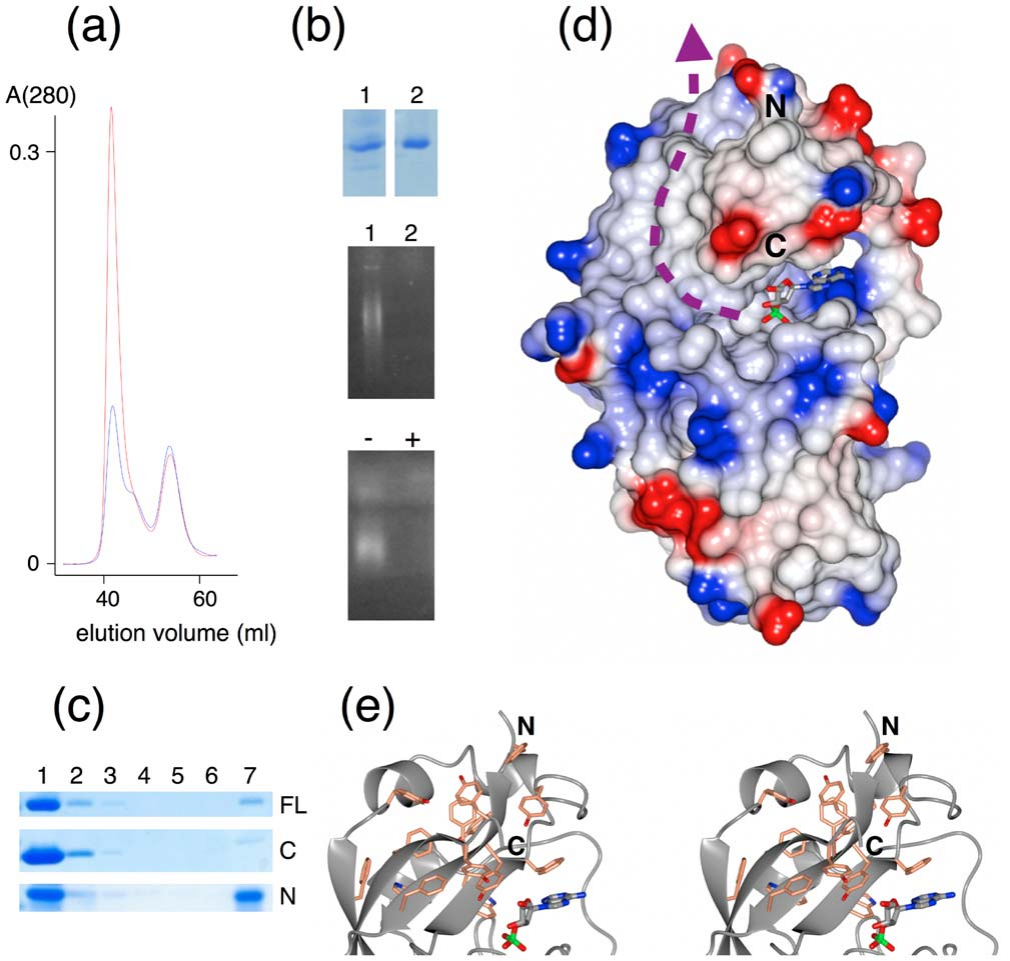
RNA binding. (a) Elution profiles of purified full-length CNPase with (blue) and without (red) RNase A treatment. (b) Gel electrophoresis of fractions from size exclusion chromatography. Top: SDS-PAGE analysis of the void volume (1) and the monomer peak (2). Middle: The same samples run on an agarose gel with ethidium bromide staining. Bottom: A sample from the void volume with (+) and without (−) RNase treatment confirms the presence of RNA. (c) Binding of different CNPase constructs to a polyadenylyl affinity matrix. 1 – input sample; 2–6 – washes; 7 – bound protein eluted from the matrix with sample buffer. FL – full-length; C – catalytic domain; N – N-terminal domain. (d) The potential beginning of the RNA-binding site in CNPase. The large hydrophobic surface extends from the active site towards the N-terminal domain. The hydrophobic region is surrounded by a rim of positive electrostatic potential. The colouring is from −0.3 V (red) to +0.3 V (blue), as implemented in ccp4mg [51]. The magenta arrow indicates the suggested binding surface for RNA. The N- and C-termini for the domain are labeled. (e) Stereo view of the aromatic cluster in the small lobe.

During purification of full-length CNPase and the N-terminal domain, a large peak is always observed at the void volume of the size exclusion column. While we originally assumed the protein is aggregated, further experiments showed that this peak contains RNA bound to CNPase; the peak height can be significantly decreased with RNase A (Figure 5A). Furthermore, RNase treatment coupled with agarose gel electrophoresis showed the void volume sample contained RNA (Figure 5B). The behaviour of CNPase in these assays is highly similar to the uracil-DNA-degrading factor [32]. No peak at the void volume is observed for the catalytic domain (data not shown), which indicates the N-terminal domain of CNPase is involved in RNA binding, at least in the case of *E. coli* RNA. Previously, CNPase was shown to bind RNA *in vitro* and be copurified with poly(A)+ RNA; the catalytic domain was sufficient to bind single-stranded RNA homopolymers in a pulldown assay [18]. We also carried out a similar pulldown experiment with poly(A)-sepharose; the N-terminal domain bound the matrix efficiently, while little binding was observed for the C-terminal domain (Figure 5C).

If the terminus of an RNA molecule indeed acts as a CNPase substrate, it will bind the surface from the active site towards the N-terminal domain (Figure 5D). In 3D space, the N-terminal domain will be a continuation of the catalytic domain small lobe. A cluster of aromatic residues is found on the surface of the catalytic domain in this area, forming a patch that leads towards the N-terminal domain (Figure 5E). Furthermore, a positively charged rim is present at the edge of this aromatic surface (Figure 5D). Thus, the surface of the small lobe may provide a binding site for RNA in the vicinity of the active site, although the N-terminal domain may be required for high-affinity binding. Further studies will be required to elucidate the details of RNA binding by CNPase.

**Figure 6:**
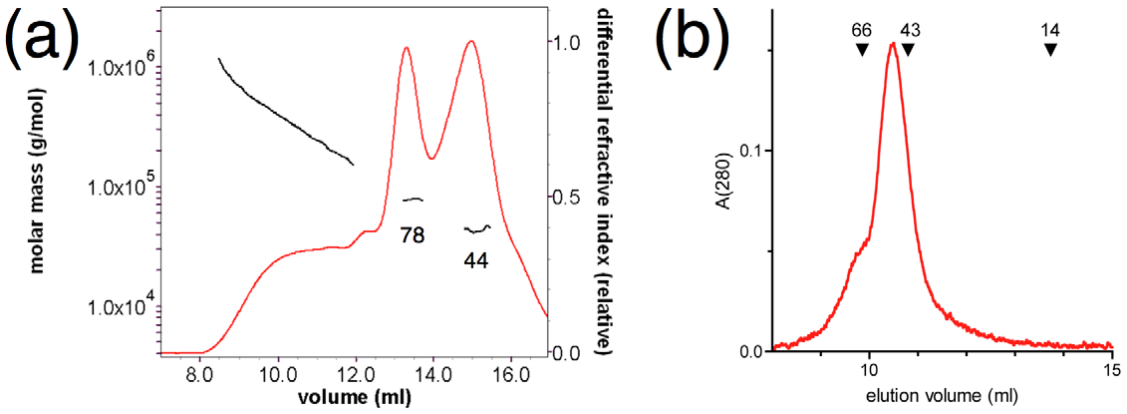
Dimerization is possibly mediated by the N-terminal domain. (a) SEC/MALS indicates monomer-dimer equilibrium for full-length CNPase. The calculated molecular masses (black) for the two peaks are 78 and 44 kDa, based on light scattering and refractive index. Higher-order oligomers are also detectable. (b) Analysis of the purified N-terminal domain by SEC indicates the presence of dimers. The elution volumes of molecular weight standards run on the same column are indicated above the graph (in kDa). The expected monomeric molecular weight is 19 kDa, and the elution volume is compatible with the presence of a slightly elongated dimer.

The tendency of self-association by full-length CNPase was also observed. As shown by a combination of size exclusion chromatography and multi-angle light scattering (SEC/MALS), the full-length protein is present as monomers, dimers, and high-molecular weight species (Figure 6A). The CNPase N-terminal domain alone elutes as a dimer on gel filtration (Figure 6B). These data strongly suggest a role for the N-terminal domain in the mediation of dimerization of full-length CNPase. No 3D structural data are available to date on full-length CNPase. The identification of the N-terminal domain as a self-association domain provides a valid explanation for early observations of putative CNPase dimers [33], [34].

### The CNPase reaction mechanism

**Figure 7:**
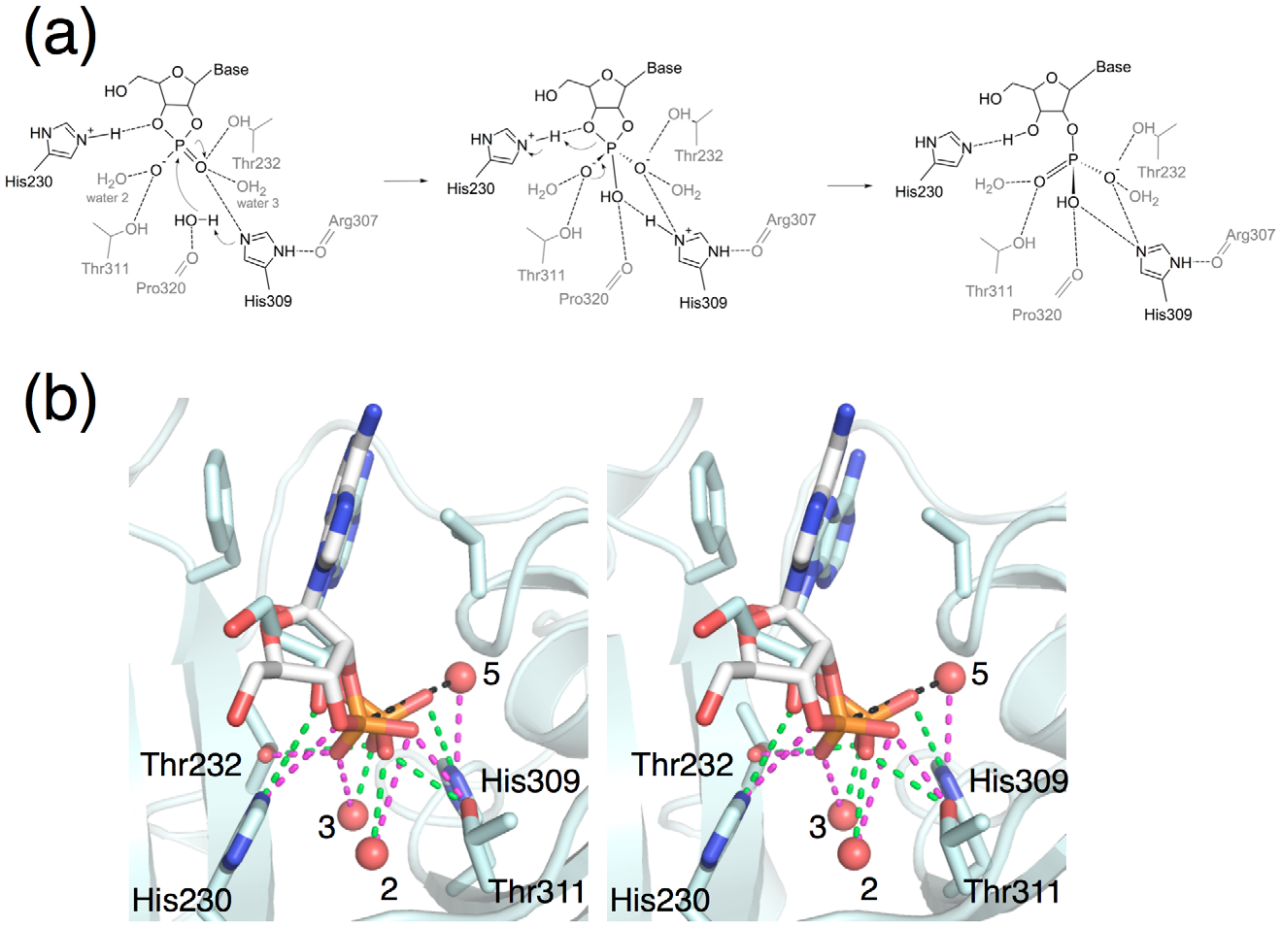
CNPase reaction mechanism, as supported by the structural data. (a) The reaction mechanism, including the H-bonding interactions in the active site. (b) Stereo view of the interactions during the reaction in the active site. Dashed lines indicate the polar interactions before (magenta) and after (green) the reaction. The black dashed line indicates the nucleophilic attack by water 5. While the product 2′-AMP (light blue) is from the crystal structure, the substrate 2′,3′-cAMP (white) was modeled into the active site by docking.

While the crystal structure of the human CNPase phosphodiesterase domain with bound phosphate [11] has enabled theories on the exact mechanism, the lack of substrate or product complexes has left uncertainties about the details of substrate/product binding. The mechanism is related to the well-characterized reaction mechanism of RNase A in its second half-reaction, where the RNA 2′,3′-cyclic end is processed to form 3′-phosphate. Mutagenesis indicated different roles for the active-site residues in CNPase; the His residues were critical for catalysis, but not substrate binding. Thr311 is more important for catalysis than Thr232, which plays a larger role in substrate binding [5]. The CNPase optimum pH (around 6) is likely to be related to the pKa values of the catalytic His residues. Our data, showing for the first time the binding mode of a physiologically relevant reaction product, now clarify the active-site geometry and interactions when nucleotide ligands are bound (Figure 7A,B). His309, oriented by the carbonyl oxygen of Arg307, activates a water molecule, which performs a nucleophilic attack on the cyclic phosphate. The pentavalent phosphate intermediate is stabilized by the protonated His230 binding to the leaving O3′-group oxygen, and the Thr residues and active-site waters coordinating the ‘free’ oxygens of the phosphate. His230 then protonates the leaving O3′-group. It is highly likely the same hydrogen-bonding contacts between the ligand and the active-site residues, including conserved water molecules, remain throughout the reaction (Figure 7A,B).

### Concluding remarks

We have now provided a long-sought high-resolution view into the liganded CNPase active site, which enables a better understanding of substrate binding and the catalytic mechanism. We also showed that the N-terminal domain mediates self-association of CNPase, and may function as an extended RNA-binding site. Conformational changes may be of importance in the CNPase catalytic cycle. The links between CNPase binding to its different ligands, its oligomeric status, and its phosphodiesterase activity still remain to be clarified. A major goal for future research will be the high-resolution structure determination of full-length CNPase.

## Materials and Methods

### Protein expression and purification

CNPase has 400 (CNP1) or 420 (CNP2) amino acid residues. The catalytic domain (residues 159–378; numbering for the CNP1 isoform) and the full-length (residues −1–378) CNPase constructs, lacking the 22-residue C-terminal tail, were overexpressed and purified as described [35]. For SAXS studies on the effects of extensions on the catalytic domain structure, 3 more constructs were used: N-cat (residues 138–378), cat-C (residues 159–400), and N-cat-C (residues 138–400). Briefly, mouse CNPase cDNA was cloned into the pTH27 expression vector [36], overexpressed in *E.coli*, and purified using nickel affinity and size exclusion chromatography, and the His-tag was removed. The phosphodiesterase activity and proper folding of the enzyme were also verified [35]. Molecular weights of full-length CNPase were determined using SEC/MALS. For the latter, an online system consisting of the Äkta Purifier (GE Healthcare), a size exclusion column (Sephadex S-200; GE Healthcare), the miniDAWN TREOS light scattering detector (Wyatt), and the Optilab rEX refractive index detector (Wyatt) was used; data analysis was performed with the Astra software (Wyatt). The running buffer was 20 mM Bis-Tris-HCl pH 5.5, 300 mM NaCl, 1% glycerol, and 1 mM DTT.

For the expression of the N-terminal domain of murine CNPase, an optimized protocol was developed. The CNPase residues −1–160 [35] were cloned into the pHMGWA expression plasmid using Gateway cloning [37]. Protein was expressed in Rosetta (DE3) cells (Novagen), with a 48-h expression at +20°C in ZYM-5052 autoinduction medium [38]. Initial purification was done using Amylose resin (NEB) in 50 mM Tris-HCl pH 9, 0.5 M NaCl, 10% (v/v) glycerol, and 1 mM DTT. The fusion protein was eluted with the same buffer containing 10 mM *D*-maltose. The maltose binding protein fusion was cleaved from CNPase by TEV protease [39]; TEV and the tag were separated from CNPase using a His-Trap Ni column (GE Healthcare) with the same buffers as above, except that the elution buffer contained 0.5 M imidazole instead of maltose. The final purification step was SEC with a Superdex 75 16/60 column (GE Healthcare) in a running buffer containing 10 mM CAPS pH 10, 0.2 M NaCl, and 1 mM DTT.

SEC analysis of the purified CNPase N-terminal domain was done using the Duoflow chromatography system (Bio-Rad) and a Superdex 75 10/300 GL column (GE Healthcare), in a running buffer containing 20 mM Na-HEPES pH 8, 200 mM NaCl, and 1 mM DTT. 250 µl of the N-terminal domain at 1.7 mg/ml were injected into the column; molecular weight markers were run under the same conditions.

### X-ray crystallography and structure analysis

For crystallization, the mouse CNPase catalytic domain was dialyzed into a buffer containing 10 mM sodium citrate pH 5.5, 50 mM NaCl, 10% glycerol, and 1 mM DTT. The detailed crystallization, soaking, and cryoprotection conditions are in Table 1. X-ray diffraction data were collected at synchrotron radiation beamlines BL14.1 (BESSY, Berlin) and I911-2 (MAX-Lab, Lund), at 100 K. Data were processed and scaled using XDS [40] and XDSi [41]. Molecular replacement was done using MOLREP [42] or PHASER [43]. The initial molecular replacement model was the catalytic domain of human CNPase [11]. Later on, the best refined coordinates of mouse CNPase were always used. Structures were refined using PHENIX [44] or REFMAC [45], and models were built using Coot [46]. Simulated annealing omit maps in the absence of bound ligand were calculated in PHENIX. Processing and refinement statistics, as well as PDB accession numbers, are in Table 2.

The Molprobity server [47] was used for validation. Normal modes were calculated using the elastic network model [48]. Structural homologs were detected using PDBeFold [49] and Salami [50], and superpositions and structure analysis were carried out in Coot, ccp4mg [51], and PyMOL (http://www.pymol.org).

### Modeling

The molecular docking studies of ligands were performed with the crystal structure of CNPase. The product and water molecules in the PDB file were removed before the docking simulations. However, crucial water molecules forming hydrogen bonds with the product in the crystal structure were retained. The structures of docked ligands were obtained from the PDB and modified if necessary. The docking studies were performed with AutoDock Vina [52], with the aid of the AutoDock Tools interface. The side chains of several residues in the binding pocket of CNPase were set to be flexible. All rotatable bonds in the ligands were allowed to rotate freely. The grid map was centered on the binding pocket of the protein, and its size was set to 21×14×18 Å. The results were analyzed using PyMOL, and the final results were selected based on the binding modes [52].

### Small-angle X-ray scattering

All samples used for SAXS eluted from size exclusion chromatography as single symmetric peaks, corresponding to monomeric forms of the CNPase catalytic domain (see [35]). Initial synchrotron SAXS data were collected on the EMBL beamline ×33 at DESY, Hamburg, Germany. The CNPase catalytic domain was first transferred by size exclusion chromatography into a buffer containing 50 mM Bis-Tris-HCl pH 5.5, 100 mM NaCl, 3% glycerol, and 1 mM DTT. SAXS data were measured at 5–15 mg/ml. For analyzing the effects of citrate on the protein, SAXS data were collected on the ID14-3 beamline at ESRF, Grenoble. The buffer consisted of 20 mM Bis-Tris (pH 5.5), 300 mM NaCl, 1% glycerol, 1 mM TCEP, and concentrations between 1–10 mg/ml were used. Data were measured in the presence and absence of 50 mM sodium citrate. The comparison of the catalytic domain with N- and C-terminal extensions was carried out on the P12 EMBL BioSAXS beamline (PETRA III, DESY, Hamburg). The samples were in 20 mM Bis-Tris (pH 5.5), 200 mM NaCl, 1 mM TCEP. Sample concentrations ranged between 3 and 15 mg/ml.

The data were analyzed with the ATSAS [53] suite, as described [54], [55]. PRIMUS was used for processing [56], GNOM for distance distribution evaluation [57], DAMMIN for *ab initio* model building [58], and DAMAVER for model averaging [59].

### Detection of bound RNA

For observing bound RNA, carried over from the expression host during affinity purification, agarose gel electrophoresis of gel filtration fractions was carried out with ethidium bromide staining. Control samples were treated with RNase A prior to electrophoresis. Purified CNPase was also subjected to RNase A treatment, followed by gel filtration on a Sephadex S-75 column (in 0.1 M sodium phosphate, 0.1 M sodium citrate, 1 M NaCl, 10% glycerol, 50 mM (NH_4_)_2_SO_4_, 1 mM DTT, pH 5.5).

Polyadenylyl sepharose matrix was prepared using CNBr-activated Sepharose 4B and polyadenylic acid (GE Healthcare), according to the protocol provided by the manufacturer. Prior to the binding assay, the matrix was equilibrated with binding buffer (10 mM Tris-HCl pH 7.5, 50 mM NaCl, 2.5 mM MgCl_2_, 1 mM DTT), adapted from [18]. The binding of various CNPase constructs to the poly(A) matrix was carried out as follows. Approximately 250 µg of each protein in a volume of 0.5 ml (in binding buffer) were mixed with 50 µl of the affinity matrix for 2 h at +4°C. The matrix was collected by centrifugation, and washed 5 consecutive times with 0.5 ml of binding buffer. Thereafter, bound proteins from the matrix, resuspended in 100 µl of binding buffer, were eluted with SDS-PAGE sample buffer and analyzed by SDS-PAGE and Coomassie staining.

## Supporting Information

Figure S1 **Stereo views of electron densities for the active-site nucleotide ligands.** Top: 2′-AMP; bottom: NADP^+^. The final refined 2F_o_-F_c_ maps (light blue) are contoured at 1.2 σ for 2′-AMP and 1 σ for NADP^+^. Simulated annealing omit maps (black) are also shown at 2.5 σ.
(TIF)

Figure S2 **Structure-based sequence alignment of mouse CNPase with other known structures from the 2H family.** Group 1 contains CNPases and RICH, and group 2 more distant homologues. The HxTx motifs are indicated by green asterisks below the alignment. The secondary structure elements are those seen in mouse CNPase (see also Figure 1).
(TIF)

Figure S3 **A major normal mode for the CNPase catalytic domain, corresponding to an open/close movement of the catalytic cleft between the two lobes.** The conformation in the crystal structure is in red, the most open conformation in magenta, and the most closed in blue.
(TIF)

Figure S4 **Lack of aggregation in the samples and concentration dependency of SAXS measurement.** The figures on the left show the Guinier plots, indicating no aggregation, while the figures on the right show the behaviour of the samples as a function of protein concentration. (a) Comparison of the CNPase catalytic domain in the presence (filled symbols) and absence (open symbols) of citrate. For the concentration dependence, radii of gyration are indicated in black and forward scattering intensities in red. (b) Analysis of N- and C-terminal extensions to the CNPase catalytic domain. Colouring as in Fig. 4B.
(TIF)
