# Supplementary figures and images for: Myelin 2′,3′-Cyclic Nucleotide 3′-Phosphodiesterase: Active-Site Ligand Binding and Molecular Conformation
Source: PLoS One. 2012 Feb 29;7(2):e32336. doi: 10.1371/journal.pone.0032336 (PMC3290555; doi:10.1371/journal.pone.0032336)

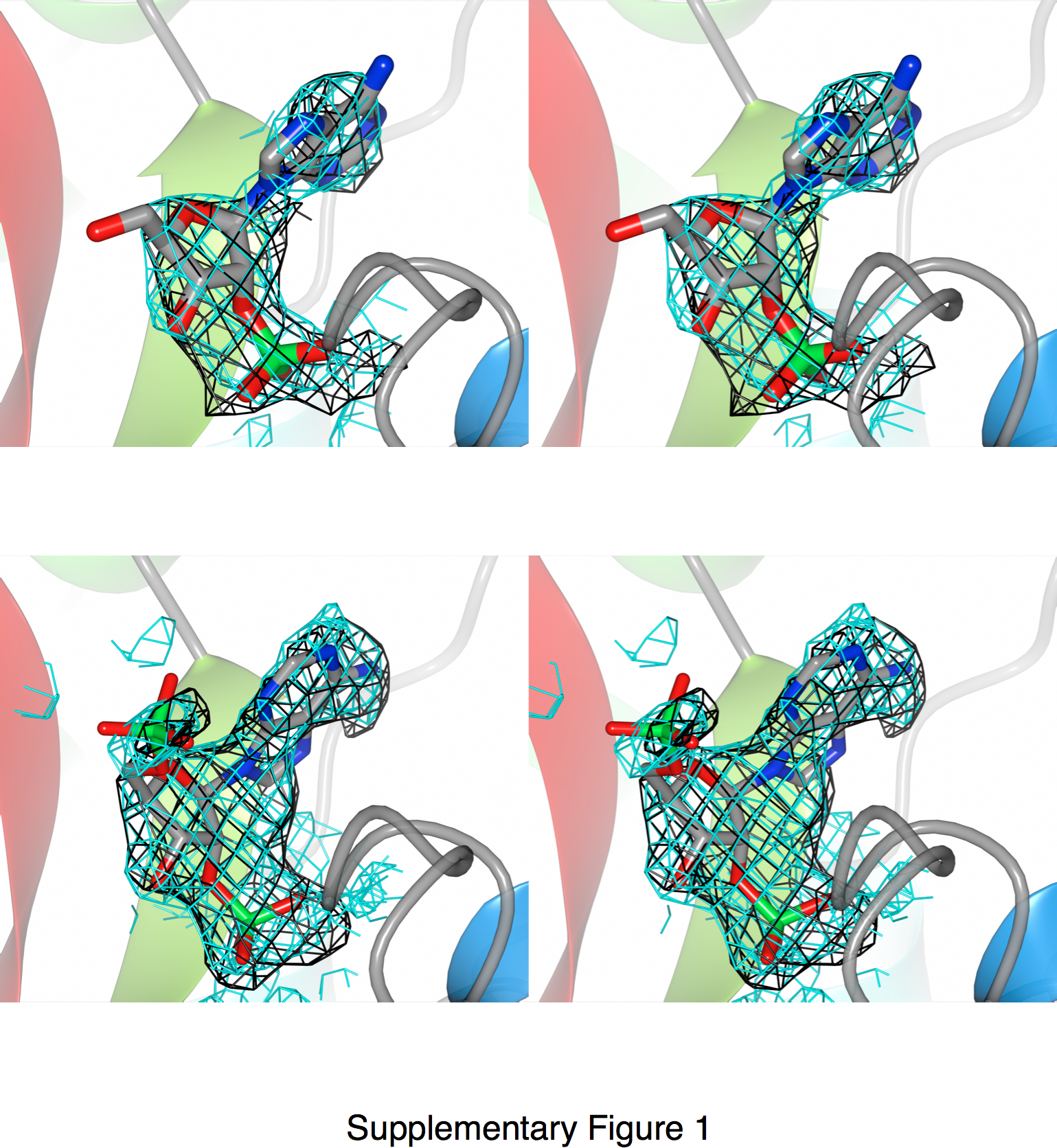

Supplement: Figure S1 — Stereo views of electron densities for the active-site nucleotide ligands. Top: 2′-AMP; bottom: NADP+. The final refined 2Fo-Fc maps (light blue) are contoured at 1.2 σ for 2′-AMP and 1 σ for NADP+. Simulated annealing omit maps (black) are also shown at 2.5 σ. (TIF) [file pone.0032336.s001.tif]

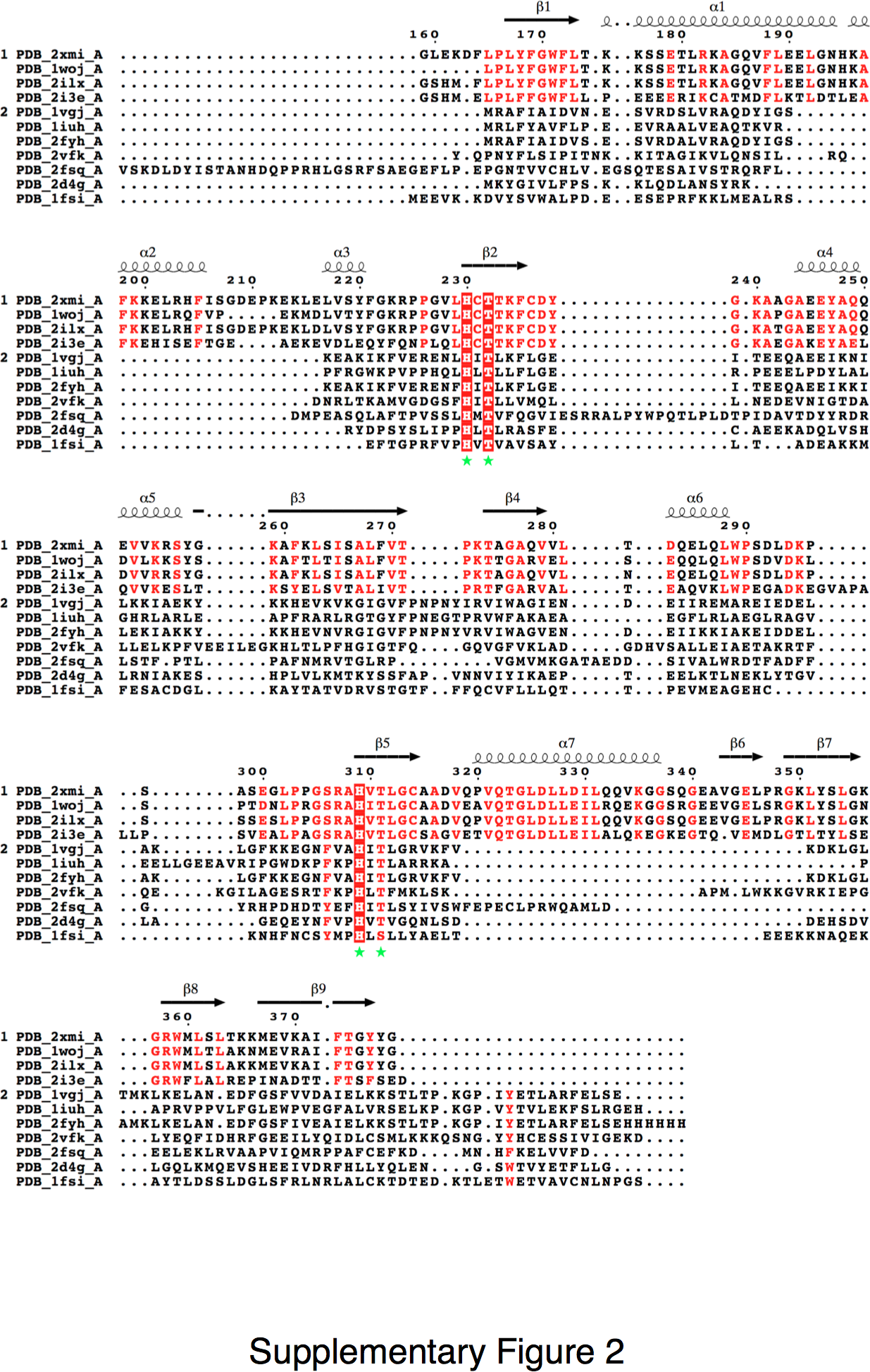

Supplement: Figure S2 — Structure-based sequence alignment of mouse CNPase with other known structures from the 2H family. Group 1 contains CNPases and RICH, and group 2 more distant homologues. The HxTx motifs are indicated by green asterisks below the alignment. The secondary structure elements are those seen in mouse CNPase (see also Figure 1). (TIF) [file pone.0032336.s002.tif]

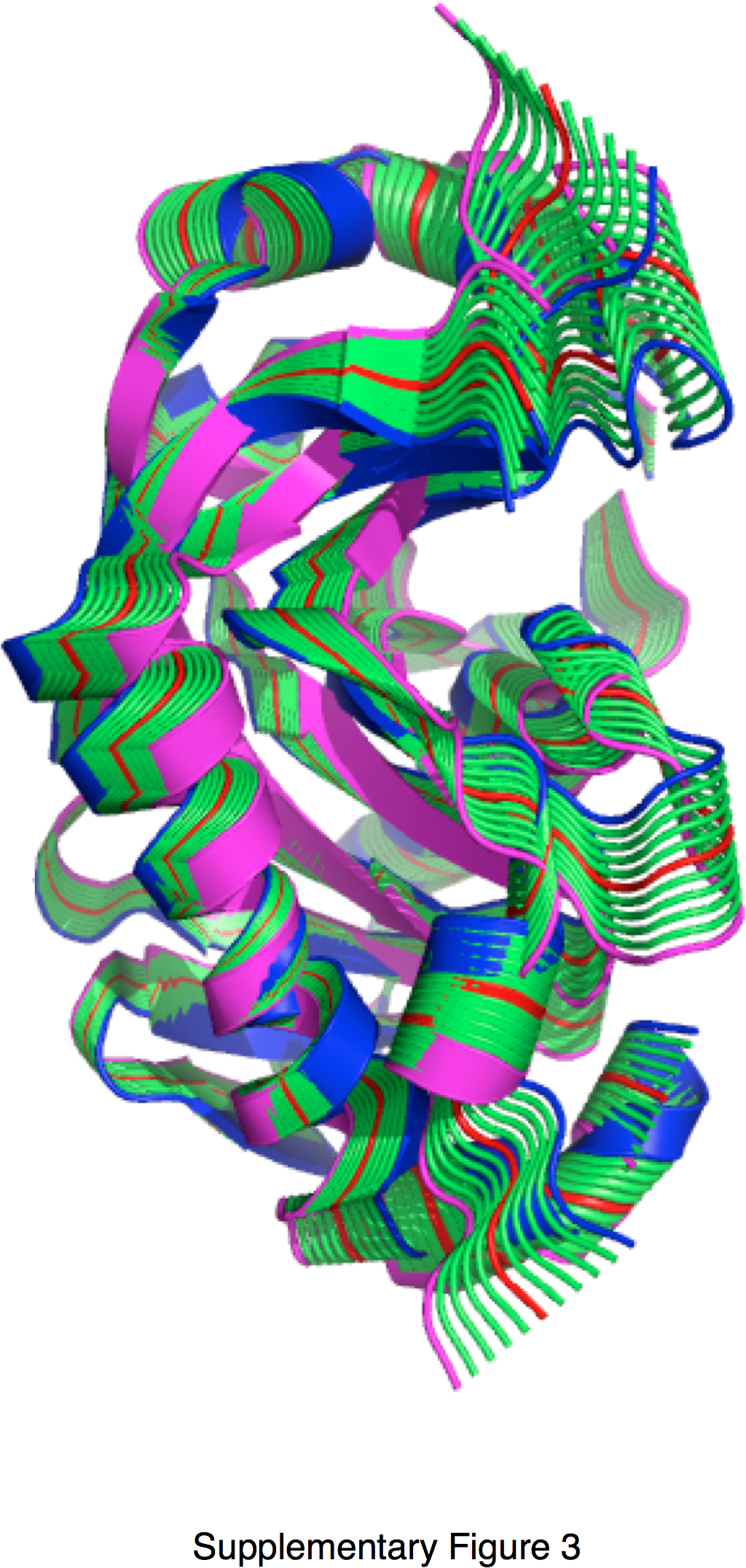

Supplement: Figure S3 — A major normal mode for the CNPase catalytic domain, corresponding to an open/close movement of the catalytic cleft between the two lobes. The conformation in the crystal structure is in red, the most open conformation in magenta, and the most closed in blue. (TIF) [file pone.0032336.s003.tif]

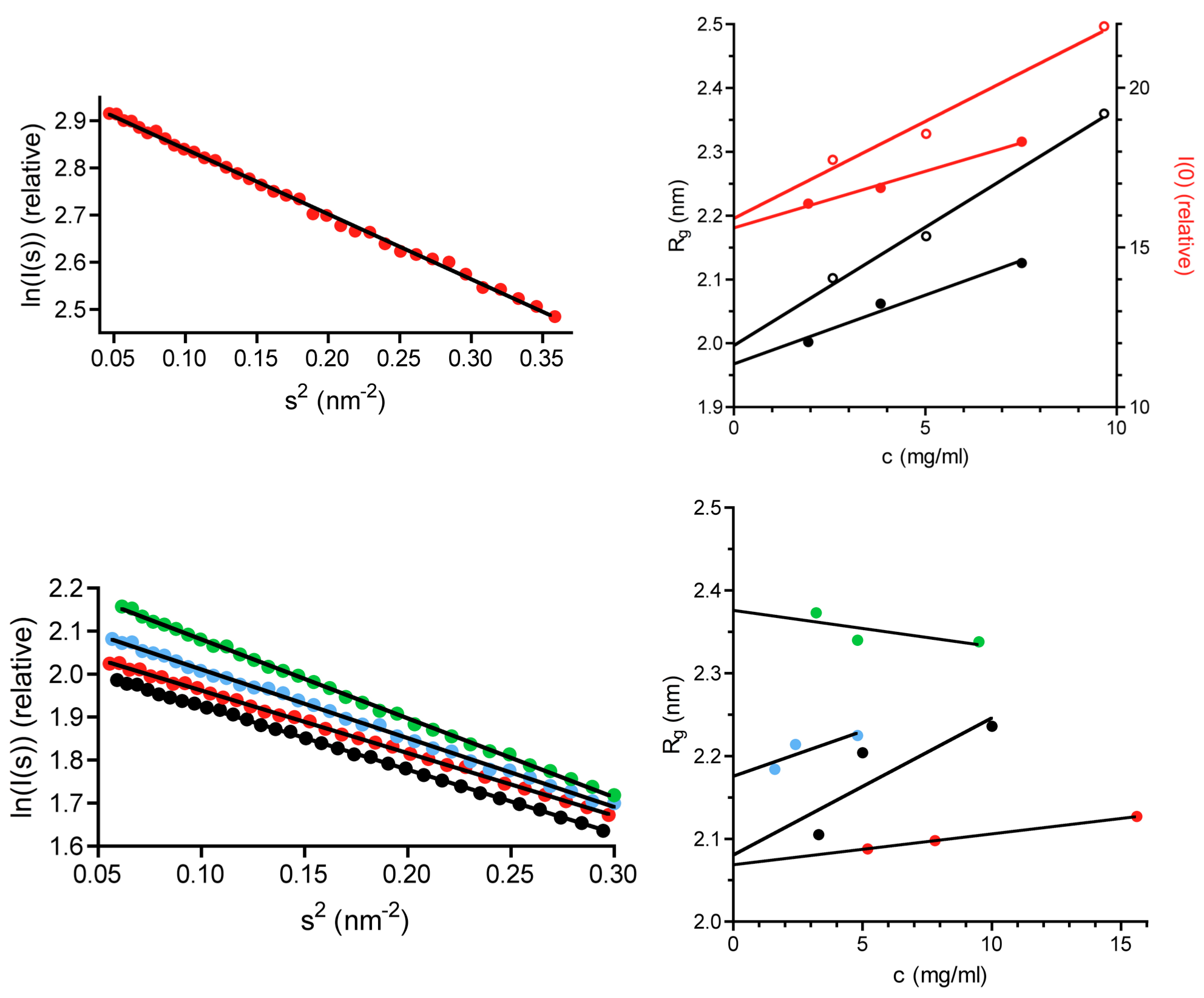

Supplement: Figure S4 — Lack of aggregation in the samples and concentration dependency of SAXS measurement. The figures on the left show the Guinier plots, indicating no aggregation, while the figures on the right show the behaviour of the samples as a function of protein concentration. (a) Comparison of the CNPase catalytic domain in the presence (filled symbols) and absence (open symbols) of citrate. For the concentration dependence, radii of gyration are indicated in black and forward scattering intensities in red. (b) Analysis of N- and C-terminal extensions to the CNPase catalytic domain. Colouring as in Fig. 4B. (TIF) [file pone.0032336.s004.tif]
